# Systematic Review with Meta-Analysis: Endoscopic and Surgical Resection for Ampullary Lesions

**DOI:** 10.3390/jcm9113622

**Published:** 2020-11-10

**Authors:** Christian Heise, Einas Abou Ali, Dirk Hasenclever, Francesco Auriemma, Aiste Gulla, Sara Regner, Sébastien Gaujoux, Marcus Hollenbach

**Affiliations:** 1 Department of Medicine I—Gastroenterology, Pulmonology, Martin-Luther University Halle-Wittenberg, 06097 Halle, Germany; christian.heise@uk-halle.de; 2 Department of Gastroenterology, Digestive Oncology and Endoscopy, Cochin Hospital, Paris Descartes University, 75014 Paris, France; einas.abouali@gmail.com; 3 Institute for Medical Informatics, Statistics and Epidemiology (IMISE), University of Leipzig, 04103 Leipzig, Germany; dirk.hasenclever@medizin.uni-leipzig.de; 4 Digestive Endoscopy Unit, Division of Gastroenterology, Humanitas Clinical and Research Hospital, Rozzano, 20089 Milan, Italy; francesco.auriemma.1987@gmail.com; 5 Department of Abdominal Surgery, Faculty of Medicine, Institute of Clinical Medicine, Vilnius University, 01513 Vilnius, Lithuania; aistegulla@gmail.com; 6 General Surgery, MedStar Georgetown University Hospital, Washington, DC 20007, USA; 7 Department of Clinical Sciences Malmö, Section for Surgery, Lund University, 221 00 Lund, Sweden; sara.regner@med.lu.se; 8 Department of Pancreatic and Endocrine Surgery, Pitié-Salpetriere Hospital, Médecine Sorbonne Université, 75000 Paris, France; sebastien.gaujoux@aphp.fr; 9 Medical Department II, Division of Gastroenterology, University of Leipzig Medical Center, 04103 Leipzig, Germany

**Keywords:** ampullectomy, papillectomy, pancreaticoduodenectomy, trans-duodenal ampullectomy, ampulla of Vater

## Abstract

Ampullary lesions (ALs) can be treated by endoscopic (EA) or surgical ampullectomy (SA) or pancreaticoduodenectomy (PD). However, EA carries significant risk of incomplete resection while surgical interventions can lead to substantial morbidity. We performed a systematic review and meta-analysis for R0, adverse-events (AEs) and recurrence between EA, SA and PD. Electronic databases were searched from 1990 to 2018. Outcomes were calculated as pooled means using fixed and random-effects models and the Freeman-Tukey-Double-Arcsine-Proportion-model. We identified 59 independent studies. The pooled R0 rate was 76.6% (71.8–81.4%, I^2^ = 91.38%) for EA, 96.4% (93.6–99.2%, I^2^ = 37.8%) for SA and 98.9% (98.0–99.7%, I^2^ = 0%) for PD. AEs were 24.7% (19.8–29.6%, I^2^ = 86.4%), 28.3% (19.0–37.7%, I^2^ = 76.8%) and 44.7% (37.9–51.4%, I^2^ = 0%), respectively. Recurrences were registered in 13.0% (10.2–15.6%, I^2^ = 91.3%), 9.4% (4.8–14%, I^2^ = 57.3%) and 14.2% (9.5–18.9%, I^2^ = 0%). Differences between proportions were significant in R0 for EA compared to SA (*p* = 0.007) and PD (*p* = 0.022). AEs were statistically different only between EA and PD (*p* = 0.049) and recurrence showed no significance for EA/SA or EA/PD. Our data indicate an increased rate of complete resection in surgical interventions accompanied with a higher risk of complications. However, studies showed various sources of bias, limited quality of data and a significant heterogeneity, particularly in EA studies.

## 1. Introduction

Ampullary lesions (AL) are rare conditions and comprise 7 to 10% of periampullary lesions [1]. However, the incidence of ALs has clearly increased from 1973 to 2005 particularly in patients over the age of 50 [2]. Nowadays, most ALs are incidentally diagnosed by endoscopy or radiology imaging. However, in symptomatic patients, painless jaundice and cholangitis, acute pancreatitis, nausea, vomiting or weight loss have been described [3]. Most ALs are ampullary adenomas (AA) and adenocarcinomas (AC) following an adenoma-to-carcinoma sequence [4]. Some other rare entities such as neuroendocrine or mesenchymal lesions have also been described [5].

Treatment of ALs is historically a surgical approach but advances in endoscopic techniques have facilitated minimally invasive therapies [6]. Non-invasive ALs, carcinoma in situ and T1-node-negative adenocarcinoma can be treated either by endoscopic ampullectomy or papillectomy (EA) [7], surgical or trans-duodenal ampullectomy (SA) [8] or pancreaticoduodenectomy (PD) [9]. However, clear consensus guidelines or recommendations are lacking and the therapeutic strategy for ALs depends on local expertise. EA is usually performed for smaller lesions without any sign of invasive carcinoma, clear margins, soft tissue and absence of ulceration [10]. In contrast, recent studies describe the feasibility of “piece-meal” EA [11], even in large laterally spreading lesions, with deep ductal invasion [12] and supposed node-negative T1 adenocarcinoma [13]. Additionally, EA could be used as a “macrobiopsy” for tumor staging and as a bridge to surgery [14]. This is important, as recent studies still show limited pre-interventional accuracy of the endoscopic biopsy despite the use of EUS [15]. In addition, the significant morbidity and mortality of PD, as well as a notable recurrence of up to 43% in EA, have to be considered in the therapeutic decision for resection of ALs [16].

To date, randomized controlled trials that have matched EA and SA or PD directly are lacking. Only a few studies retrospectively compared endoscopic and surgical techniques for the treatment of ALs. These works revealed different inclusion criteria, outcomes and surgical approaches. In addition, the conclusions drawn were, at least in part, counterintuitive [11,16,17]. Thus, the aim of this systematic review and meta-analysis was to analyze the outcomes of EA, SA and PD for non-invasive ALs, carcinoma in situ and T1-node-negative adenocarcinoma in terms of complete resection (R0), complications and recurrences.

## 2. Methods

### 2.1. Data Sources

Our search strategy and inclusion criteria were based on the Preferred Reporting Items for Systematic Reviews and Meta-Analyses (PRISMA) recommendations [18]. The analysis was registered in the PROSPERO-Database (on 19 August 2019, registration number 147795). We performed a systematic literature reveiw in Medline, EMBASE and SCOPUS on 27 September 2018 to identify studies that analyzed endoscopic or surgical interventions for ALs. Additionally we performed a manual research of references from representative articles and reviews.

### 2.2. Study Selection Process and Outcome Measures

Only human subject studies were considered in the analysis. Any retrospective or prospective study analyzing EA, SA or PD for ALs that reported at least one of the outcomes was included. The primary outcome measurement of our study was the rate of complete resection (R0) determined by histology. Secondary outcomes were overall complications (AEs) for endoscopic and surgical interventions and rate of recurrence. Recurrence was defined as a new lesion on endoscopy after initial negative follow-up endoscopy (in EA and SA) or local or distant recurrence in cross-sectional imaging (SA and PD). Further inclusion criteria were histologically proven ALs, patients >18 years, >10 patients per study, >90% ampullary adenoma or adenocarcinoma (T1 or carcinoma in situ), publication later than 1990. Studies were excluded, if they were deemed to have insufficient data or did not report independent data (e.g., review articles, editorials, correspondence letters, duplication and others) or did not fulfil inclusion criteria. A bibliography of full texts was established for all studies that could possibly meet inclusion criteria.

### 2.3. Quality Assessment

The data quality of the included studies was evaluated by the modified Newcastle–Ottawa Scale (NOS) (Table 1) for non-randomized studies, ranging from 0 (low-quality) to 9 (high-quality) [19]. The overall NOS-Score was calculated by 7 items (4 points for adequate selection, 2 for comparability and 3 for outcome). A study was scored as good quality if at least 3 points for selection, 1 for comparability and 2 for outcome were achieved. Fair quality was defined as 2 points for selection, 1 point for comparability or 1 point for outcome. Studies of poor quality reached 1 point for selection, no points for comparability or 1 point in outcome. All reviewers (CH, EAA, FA, AG, MH) assessed the specific quality indicators of the included publications. 

### 2.4. Statistical Analysis

Statistical analyses were performed by means of openMeta[Analyst] (v. 12.11.14, http://www.cebm.brown.edu/openmeta/), “R” (version 3.5.3 distributed by www.r-project.org) and SPSS (version 25, IBM). We calculated pooled values for R0, complications and recurrence and presented the proportion with a 95% confidence interval (CI). The binary random effects model (DerSimonian–Laird) was used for analysis of EA and SA, as these studies showed high heterogeneity. In contrast, meta-analyses for PD were calculated by a fixed effect model with inverse variance weighting as data were of low heterogeneity. Statistical heterogeneity between the included studies was determined by forest plots and by calculating the I^2^ index. In the case of high heterogeneity, a sensitivity analysis was performed. All pooled event rates were shown in forest plots, regardless of the level of heterogeneity.

Studies were evaluated for publication bias by Egger’s test [20], and funnel plots were drawn by using JASP (v. 0.11.1, https://jasp-stats.org/). To compare endoscopic and surgical interventions, a proportion-meta-analysis was conducted. The individual study proportions were transformed into a quantity using the Freeman–Tukey Double Arcsine Proportion model. Then, the pooled proportion was calculated by transforming the weighted mean back out of the transformed proportions. For the transform, we used inverse arcsine variance weights for the fixed-effects model and DerSimonian–Laird weights for the random effects model [21]. Statistical significance was assessed by considering the 95% CIs of the two pooled proportions and the differences of proportions and their 95% CIs, respectively. All calculated *p*-values by Students *t*-test were 2-sided, and *p* < 0.05 was considered statistically significant. Combined weighted proportions were determined by use of the openMeta[Analyst].

Search strategy, screening and data extraction can be found in the supplemental material in methods.

## 3. Results

### 3.1. Study Characteristics and Quality

**Figure 1:**
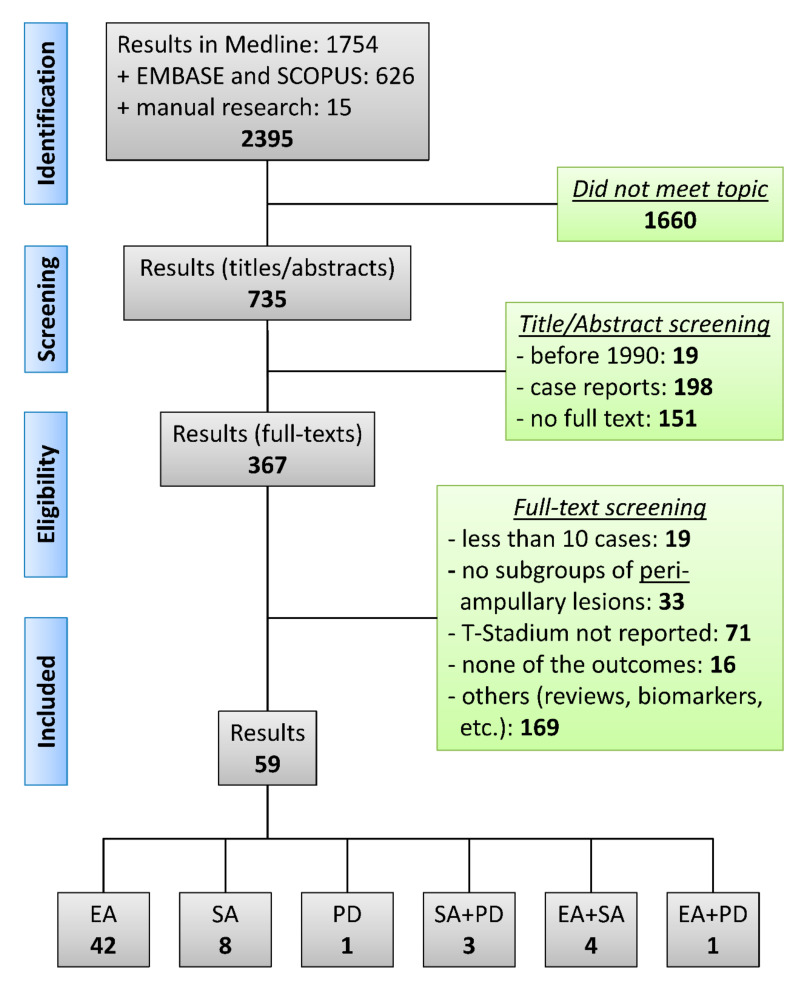
Flow chart of the study selection process. Out of 2395 search results, 59 papers were finally included in the analysis. EA: endoscopic ampullectomy, SA: surgical ampullectomy, PD: pancreaticoduodenectomy.

Our literature search revealed 2395 articles in total. Out of these, 1660 did not meet the topic. After screening of titles and abstracts, 367 studies remained. Finally, 59 papers were eligible to be included into the meta-analysis after full-text evaluation (42 EA, 8 SA, 1 PD, 3 SA + PD, 4 EA + SA, 1 EA + PD; Figure 1 PRISMA chart) [22,23,24,25,26,27,28,29,30,31,32,33,34,35,36,37,38,39,40,41,42,43,44,45,46,47,48,49,50,51,52,53,54,55,56,57,58,59,60,61,62,63,64,65,66,67,68,69,70,71,72,73,74,75,76,77,78,79]. We did not include conference abstracts that were not published as full-texts. Although some studies reported outcomes of different interventions, no single study directly compared different approaches with relevant patient count. Relevant study characteristics and quality assessment are reported in Table 2; Table 3. The majority of studies (*n* = 54, 92%) were retrospective single center reports. In total, out of the EA studies, we included 2658 patients (54.1% male) at an average age of 59.2 years for our analysis. Moreover, SA studies involved 393 patients (47.5% male) with a mean age of 63.5 years and PD studies involved 778 patients (53.3% male) with a mean age of 63.9 years. The follow-up after the procedures ranged between 5 and 190 months but was not adequately described in 18 studies (31%, Table 3).

The quality of the included articles was heterogeneous. Out of 59 papers, 35 were of good quality (59.4%) and 23 revealed poor scientific quality (38.9%) according to the NOS. One study was of fair quality (1.7%, all Table 2). Study quality was included in the sensitivity analysis.

### 3.2. Complete Resection in Endoscopic and Surgical Interventions

We were able to include 39 datasets of EA, 10 datasets of SA and 4 of PD in the meta-analysis for complete resection. Patients were asymptomatic in 54.4% (EA), 35.3% (SA) and 21.6% (PD). The mean lesion size of the different study cohorts was 19.7 mm (EA), 17.5 mm (SA) and 17.0 mm (PD). T1-adenocarcinoma and carcinoma-in-situ were found in 8.7% of patients in EA, in 30.8% in SA and in 100% of patients in PD (all Table 3). The remaining patients had non-invasive ALs. Thereby, a strict comparison, matching or propensity scoring was impossible to perform.

**Figure 2:**
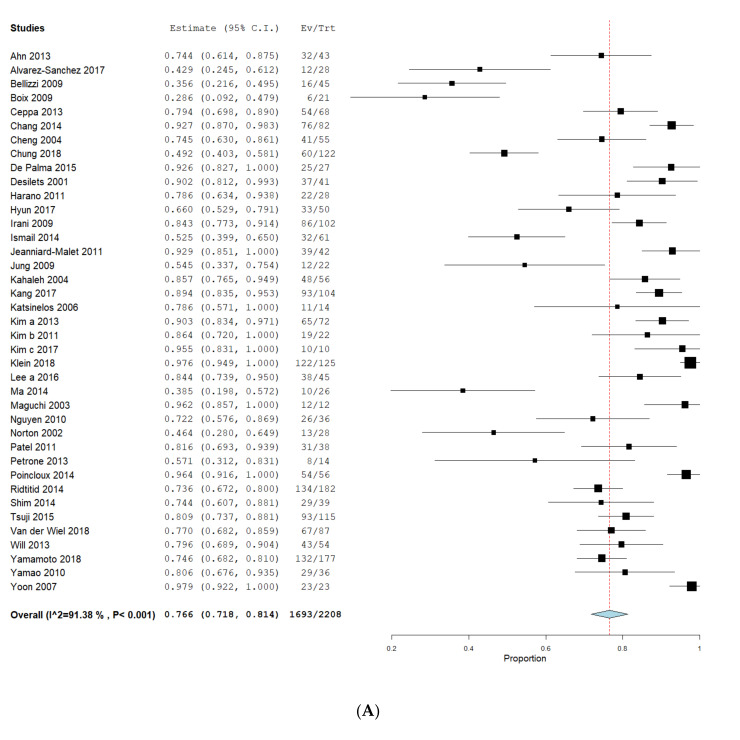

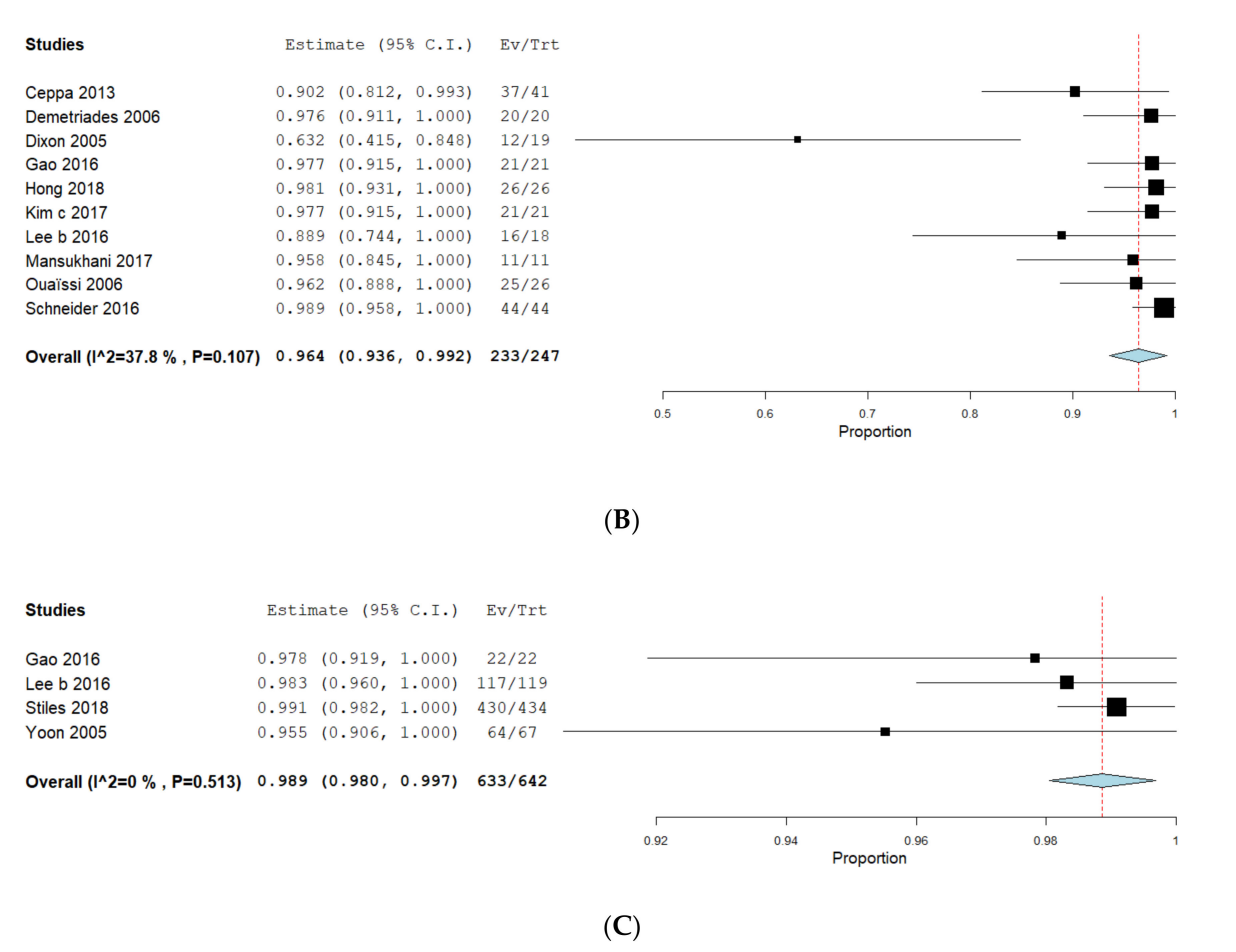
Complete resection in endoscopic and surgical intervention. Proportions of meta-analyses were calculated and shown as forrest-plots for EA (**A**), SA (**B**) and PD (**C**). Pooled R0 rates were calculated to obtain a proportion with a 95% confidence interval (CI). The binary random effects model (DerSimonian–Laird) was used for EA and SA and a fixed effect model with inverse variance weighting was used for PD. EA: endoscopic ampullectomy, SA: surgical ampullectomy, PD: pancreaticoduodenectomy.

The pooled R0 rate was 76.6% (CI 71.8–81.4%) for EA (Figure 2A), 96.4% (CI 93.6–99.2%) for SA (Figure 2B) and 98.9% (CI 98.0–99.7%) for PD (Figure 2C). Nevertheless, published data of EA (I^2^ = 91.4%, *p* < 0.001) showed high heterogeneity compared to SA (I^2^ = 37.8%, *p* = 0.107) and PD (I^2^ = 0%, *p* = 0.531, all Figure 2). The proportion-meta-analysis resulted in transformed R0-rates of 77.3% (CI 71.7–82.5%, SE 0.032, I^2^ = 88.1%, *p* < 0.001) for EA, 94.6% (CI 89.1–98.4%, SE 0.045, I^2^ = 50.5%, *p* = 0.033) for SA and 99.2% (CI 98.1–99.9%, SE 0.020, I^2^ = 0%, *p* = 0.212) for PD. Calculations showed a significant difference for complete resection of EA compared to SA (*p* = 0.007) and EA compared to PD (*p* = 0.022).

### 3.3. Complications

**Figure 3:**
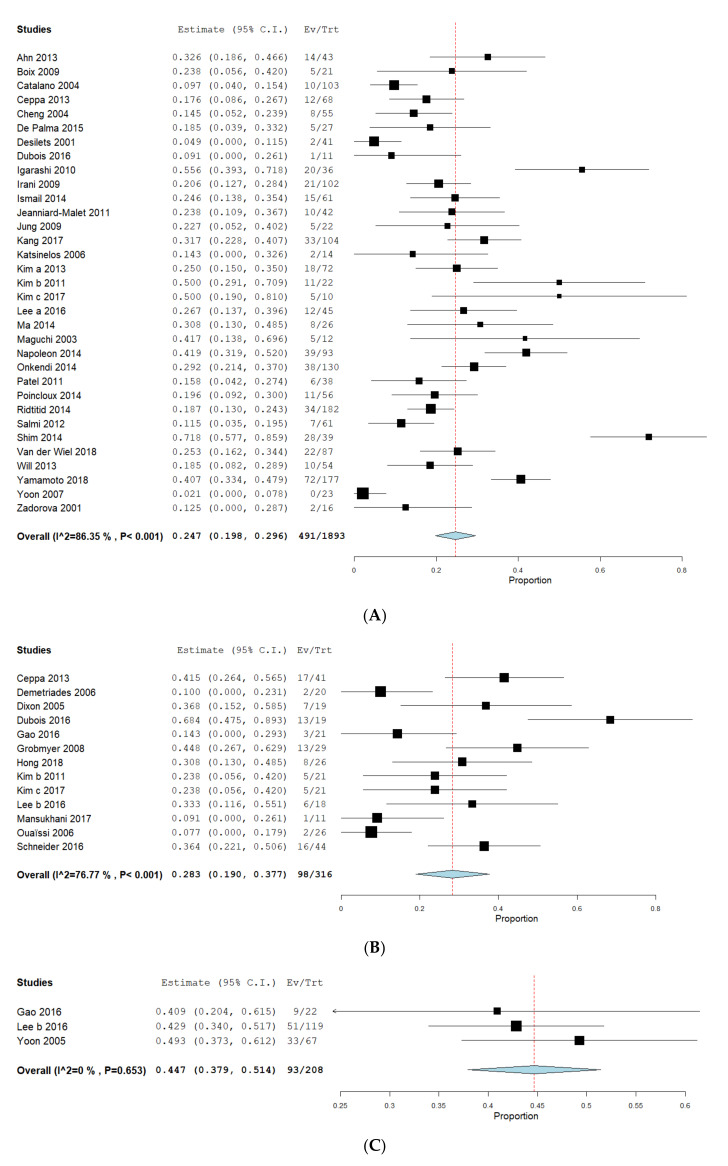
Complications in endoscopic and surgical intervention. Proportions of meta-analyses were calculated and shown as forrest-plot for EA (**A**), SA (**B**) and PD (**C**). Pooled rates of complications were calculated to obtain a proportion with a 95% confidence interval (CI). The binary random effects model (DerSimonian–Laird) was used for EA and SA and a fixed effect model with inverse variance weighting for PD. EA: endoscopic ampullectomy, SA: surgical ampullectomy, PD: pancreaticoduodenectomy, AP: acute pancreatitis, NA: not available.

Complications were assessed in 32 EA-datasets, 13 SA-datasets and 3 PD-datasets. As different types of complications were reported (Table 3), we conducted a meta-analysis to obtain a pooled estimate rate only for overall complications. Pooled overall complication rates were as follows: 24.7% (CI 19.8–29.6%) for EA (Figure 3A), 28.3% (CI 19.0–37.7%) for SA (Figure 3B) and 44.7% (CI 37.9–51.4%) for PD (Figure 3C). Heterogeneity was high in EA (I^2^ = 86.4%, *p* < 0.001) and SA (I^2^ = 76.8%, *p* < 0.001) but not PD (I^2^ = 0%, *p* = 0.653). The proportion-meta-analysis showed a pooled and transformed rate of complications of 24.5% (CI 19.8–29.4%, SE 0.027, I^2^ = 80.5, *p* < 0.001) for EA, 28.7% (CI 20.0–38.2%, SE 0.049, I^2^ = 66.5%, *p* < 0.001) for SA and 44.7% (CI 37.8–51.6%, SE 0.035, I^2^ = 0%, *p* = 0.662) for PD. The calculated difference was significant for EA compared to PD (*p* = 0.049) but not to SA (*p* = 0.524). Sensitivity analysis failed to reduce heterogeneity in the EA or SA group. We did not evaluate mortality or length of hospital stay as these data were only rarely reported. Sensitivity analysis failed to identify outliers that significantly influenced heterogeneity.

In addition, we evaluated the endoscopic publications for biliary/pancreatic duct stent implantation, submucosal injection and additional therapy that could influence the development of post-interventional complications. Thereby, papers also showed a high heterogeneity in this regard (Table 4). Sensitivity analysis failed to reduce heterogeneity of the calculated proportions as no single or systematic outliers could be identified.

### 3.4. Recurrence

**Figure 4:**
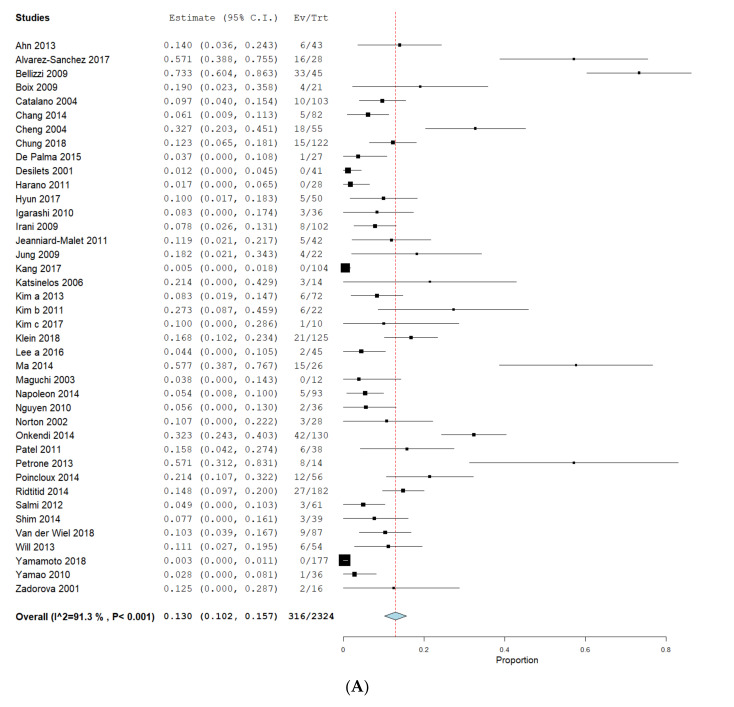

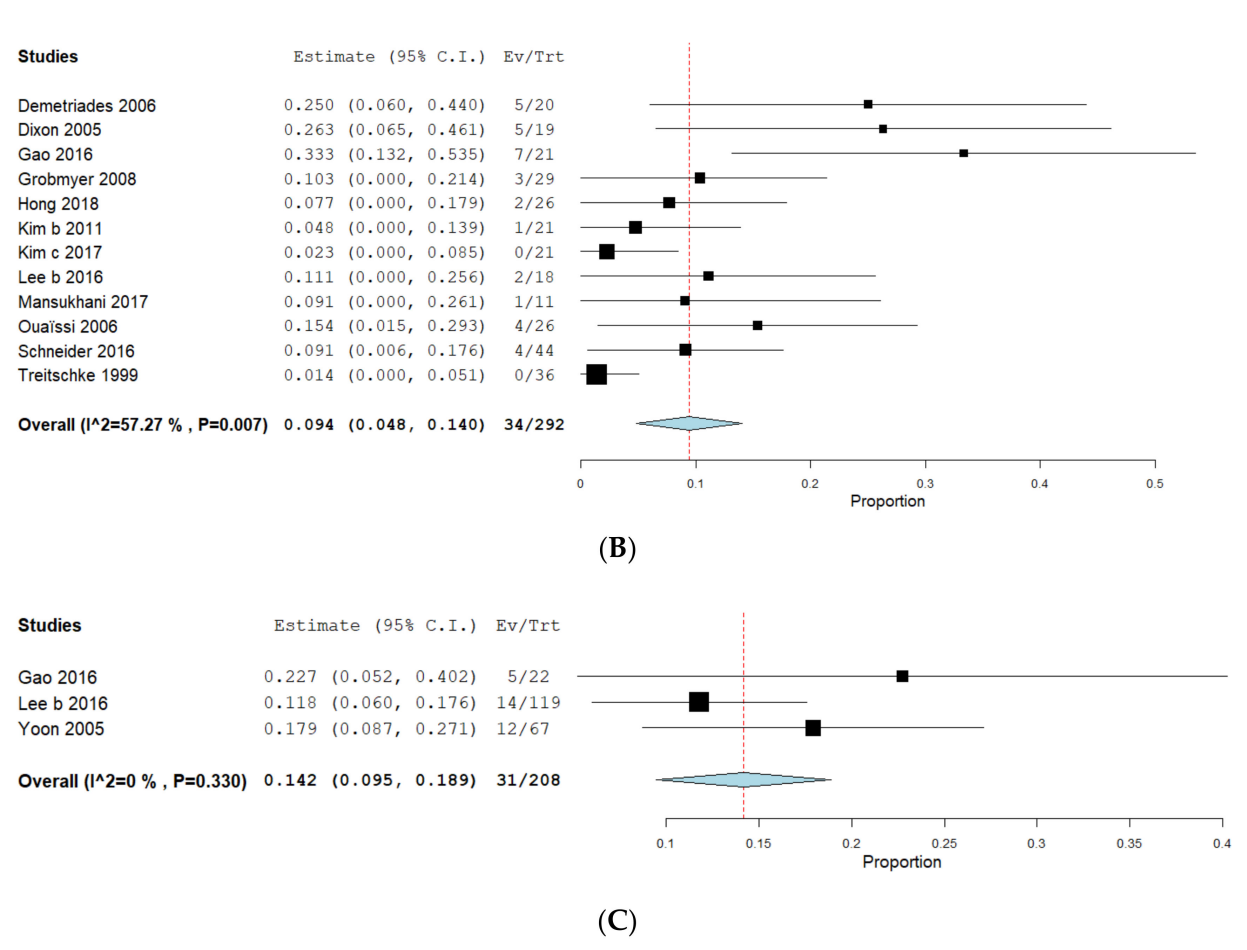
Recurrence in endoscopic and surgical intervention. Proportions of meta-analyses were calculated and shown as forrest-plots for EA (**A**), SA (**B**) and PD (**C**). Pooled rates of recurrence were calculated to obtain a proportion with a 95% confidence interval (CI). The binary random effects model (DerSimonian–Laird) was used for EA and SA and a fixed effect model with inverse variance weighting was used for PD. EA: endoscopic ampullectomy, SA: surgical ampullectomy, PD: pancreaticoduodenectomy.

In order to assess recurrence, we included data from 40 EA publications, 12 SA-datasets and 3 PD-datasets. The mean follow-up time was 44.7 months (EA), 49.6 months (SA) and 37.9 months (PD, all Table 3). The pooled means for recurrences were 13.0% (CI 10.2–15.6%) in EA, 9.4% (CI 4.8–14%) in SA and 14.2% (CI 9.5–18.9%) in PD. Heterogeneity was high in EA (I^2^ = 91.3%, *p* < 0.001) and SA (I^2^ = 57.3%, *p* = 0.007) but low in PD (I^2^ = 0%, *p* = 0.330). Proportion-meta-analysis showed pooled estimates of recurrence of 13.2% (CI 9.1–17.9%, SE 0.031, I^2^ = 88.4%, *p* < 0.001) in EA, 11.0% (CI 6.0–17.0%, SE 0.041, I^2^ = 48.9%, *p* = 0.028) in SA and 14.4% (9.8–19.7%, SE 0.035, I^2^ = 0%, *p* = 0.277) in PD (see Figure 4). Calculations revealed no statistical significance for recurrence for EA compared to SA (*p* = 0.536) or PD (*p* = 0.904).

### 3.5. Publication Bias

**Figure 5:**
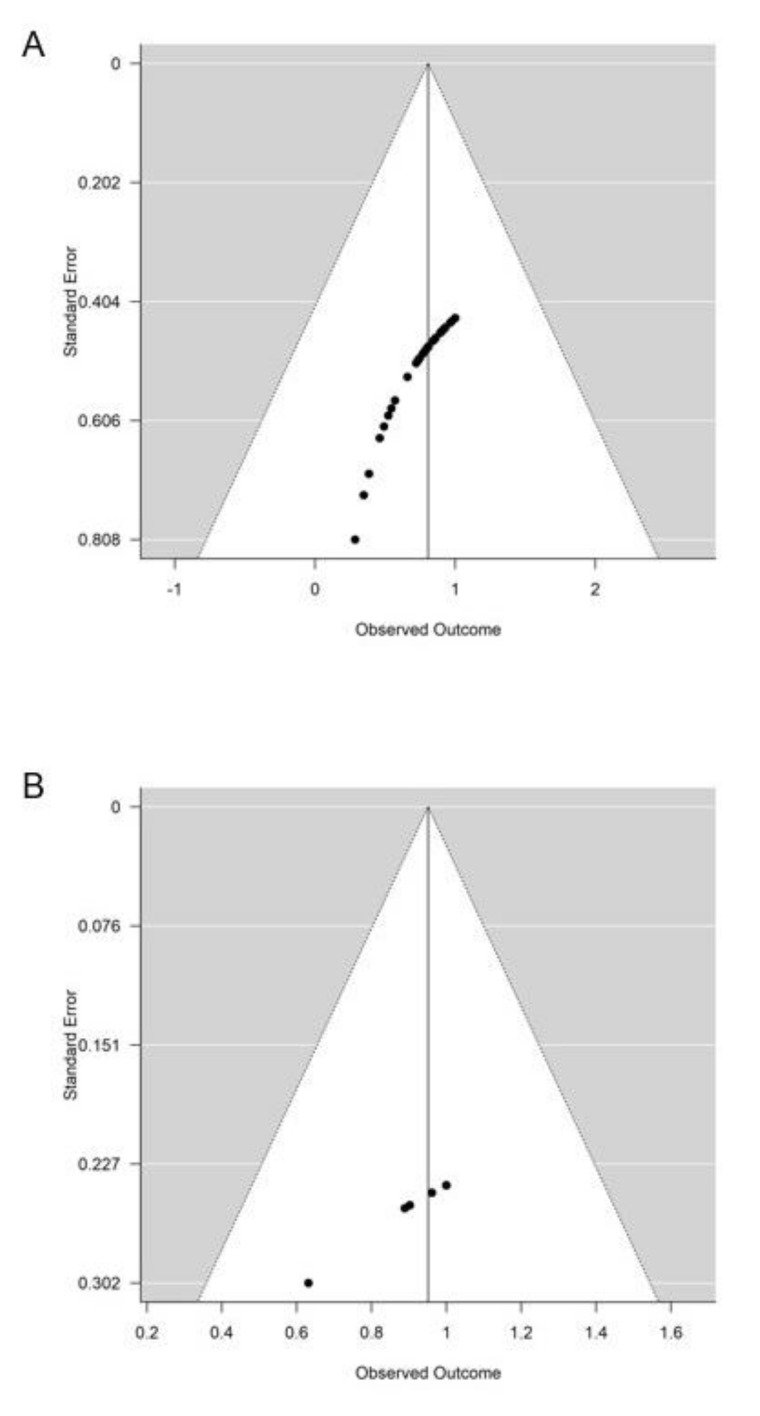
Analysis of publication bias. Funnel plots were drawn for EA (**A**) and SA (**B**) indicating no evidence of publication bias. Funnel plots were not used for PD as less than 10 PD papers were included in the meta-analysis. EA: endoscopic ampullectomy, SA: surgical ampullectomy, PD: pancreaticoduodenectomy.

The calculation of funnel plots for reported R0-rates for EA (Figure 5A) and SA (Figure 5B) showed no evidence of publication bias. The *p*-values of the Egger’s test were 0.061 for EA and 0.229 for SA. We did not analyze publication bias for PD as less than 10 PD studies were included. Our data revealed no evidence for publication bias in regard to rates of complete resection.

## 4. Discussion

ALs are a rare but increasingly diagnosed entity. Although mainly benign and premalignant lesions are diagnosed, ALs have the potential for malignant transformation. In addition, ALs can cause cholangitis or pancreatitis and thereby reduce patients’ quality of life. Thus, treatment of ALs either by EA, SA or PD is indicated in all cases [2,3,4,80]. Nevertheless, prospective randomized trials comparing EA, SA or PD are lacking and the treatment of AL for non-invasive lesions, carcinoma in situ and T1-node-negative adenocarcinoma is far from a consensus. The choice of treatment is currently based on the decision of the treating clinician, and available endoscopic or surgical resources and is not supported by evidence-based guidelines.

This study is the first systematic review to report R0 resection rate, complications and recurrence of all three interventional procedures for the treatment of ALs. Our analyses clearly indicated a superiority of surgical approaches to endoscopic therapy with regard to complete resection (EA 76.6%, SA 96.4%, PD 98.9%). Nevertheless, this high efficacy was accompanied by a considerable rate of complications, in particular for PD (EA: 24.7%, SA: 28.3%, PD: 44.7%), but is possibly under-evaluated in these retrospective single cohort series. Statistical calculations indicate a significant difference between endoscopic and surgical interventions. One could argue that there is sufficient evidence to recommend surgery for invasive ALs as first line therapy. However, for non-invasive lesions or only suspicious one, especially in patients with high risk for surgical complications, the best approach remains to be determined. In addition, there is high heterogeneity and many limitations in the available literature.

First, we had to exclude numerous studies of PD data as they provided R0-rates and complications for the whole study populations including distal bile duct carcinoma, pancreatic cancer, duodenal lesions and all stages of ALs and ACs. As a consequence, distinct data of AAs or ACs (T1) could not be assessed. Therefore, many articles that presumably would fit our analysis had to be excluded. It is nevertheless unlikely that they would have significantly modified the R0 resection rate. Finally, only four publications of PD for ALs were eligible. Hence, we cannot exclude a publication bias as the Egger’s test required a minimum of 10 publications to sufficiently address this issue. Moreover, all PD papers analyzed data of ACs but not AAs or other ALs. Unfortunately, this finding leads to the assumption of a selection bias, as ACs are preferentially treated surgically rather than endoscopically. In addition, the selection of ACs could explain the low heterogeneity of PD data due to high similarity of included patients. In contrast, ACs were found only in 8.7% of EA patients and in 30.8% of SA data, which might reflect accurate patient selection. Indeed, in T1 adenocarcinoma, only those with an intact submucosal layer (D0) are eligible for local treatment (either EA or SA) as their likelihood of negativity in lymph node invasion is very high [81,82,83]. This may also have introduced a selection bias in the published data as described above. Finally, EUS should be used to evaluate ALs prior to intervention [84] but was mentioned in only 70.6% of surgical and 65.2% of EA studies. The inequality in ACs between the three interventional groups could also explain the similar rate of recurrence, although SA and PD showed a convincing rate of complete resection. A recurrence of a completely resected AA is more unlikely compared to a T1 AC, according to its nodal status. Additionally, recurrences were not described as local or distant, which is of utmost importance. The mean follow-up after procedure was possibly too low to catch all local recurrences.

Moreover, the histologic type (intestinal vs. pancreaticobiliary) and grade of dysplasia (low grade vs. high grade vs. T1d1 invasive lesion vs. T1d2 advanced lesion) also have an impact on outcome. It is acceptable to perform a second papillectomy for an initially incomplete resected AA rather than send for surgery. In contrast, an incidentally diagnosed AC, either incompletely resected or associated with a significant risk of lymph node involvement, might require additional surgery. In addition, it is important to report the subtypes of ACs (intestinal vs. pancreaticobiliary), as they influence the risk of local or distant recurrences [85]. However, the discrimination of AC subtypes was reported in only two of all included EA studies [23,65] and in none of the surgical series. It is also important to note that EA is a recent technique that has improved over the years, and results from the 1990s or early 2000 might not be as good as today, regarding patient’s selection, the rate of R0 resection and post procedure outcomes. Another important parameter to discriminate AAs from ACs, in particular in large ALs, is the molecular markers. A KRAS-mutation was found more often in advanced AAs and in ACs [86,87]. It is nevertheless important to note that KRAS mutation is an early event in tumorigenesis and can be found in early AA. Molecular markers in ALs are not sufficient to incorporate into our analysis but are interesting for future projects.

Another limitation of our meta-analysis is based on the design of the included studies. Almost all data were published in monocentric retrospective cohort studies. Prospective comparative randomized trials are lacking. Thus, the quality of the included studies is heterogeneous, only 59.4% of papers were of good quality according to NOS. In particular, the quality of EA data remains, at least in part, limited. Technical specifications of EA procedures have developed over the time and thus, different techniques were published. Moreover, the definition of R0 by histology and/or complete endoscopic resection was not used consistently in all studies. Beside the use of EUS, a prior ERCP procedure could influence results. Many publications did not report about prior sphincterotomy and, maybe more importantly, the use of pancreatic and bile duct stents was inhomogeneous. The current ESGE guidelines clearly recommend the implantation of a pancreatic duct stent to prevent post-ERCP-pancreatitis [88,89] in EA. A lot of EA data were published prior to these guidelines and, thus, did not regularly use pancreatic duct stents. Moreover, in 40.4% of patients with EA, a prior submucosal injection was performed to lift the lesions. A submucosal injection is not recommended as such injection resulted in reduced rates of complete resection [10,31,41,80]. Furthermore, in some EA studies, additional therapy by radiofrequency ablation (RFA), argon plasma coagulation (APC), additional EA or surgery was performed. All these confounders may have substantially influenced the results, in particular for complete resection, and could explain the high heterogeneity in EA studies. However, we aimed to reduce heterogeneity by different sensitivity analyses. Unfortunately, that results remain similar (success rates did not differ >10% between different analyses).

In addition to a complete resection, we analyzed the overall rate of complications between endoscopic and surgical interventions. The rate of complications was clearly higher in PD compared to EA, but not compared to SA. Since complications are defined very specifically with respect to every interventional approach and are difficult to compare, we decided to solely compare rates of overall complications. Moreover, if overall complications were reported in the included studies, most of them were not graded according to modern classifications such as Clavien–Dindo and it was not possible to accurately classify and compare them between PDD, SA and EA according to their severity. Therefore, our analysis is limited, as we cannot judge the burden of the reported adverse events. We think that an evaluation of severity of complications ought to be examined if there is an extension of in-patient time. In addition, the postoperative mortality of PD was not assessable in the present meta-analysis but should not been underestimated, as recently shown by nationwide data analyses [90,91].

Despite all the above mentioned limitations, our meta-analysis has some strengths. The baseline characteristics of age and gender were comparable between the three groups. Interestingly, the mean size of the lesions was quite similar (EA 19.7 mm, SA 17.5 mm, PD 17.0 mm). This finding was surprising, because large lesions are often more likely to be treated by surgery. But our finding is in line with current data indicating that there is no correlation of size and malignancy, if malignancy was excluded by EUS [11,14,17,92]. Hence, a selection bias depending on the size of the lesions is very unlikely.

Our results are in line with a recently published (non-systematic) meta-analysis of five studies summarizing that surgery was more effective compared to EA in AAs, but was associated with higher rates of complications [93]. However, this analysis showed several limitations in design and statistical analysis. The current data raised several questions that need to be addressed by further studies. Even if surgery is more effective in treating AL in our meta-analysis, a risk-benefit analysis has to be performed for PD according to the high rates of AEs. Another concern is the role of SA, as it is restricted to expert centers and not widely available, and might call for centralization in the management of such rare lesions. A more fundamental issue is the long-term outcome of repeated EA for recurrent AA. There are no data comparing repetitive EA with surgery for AA or EA as bridge to surgery.

From the present meta-analysis, very few of the included studies were comparative. Additionally, because of the heterogeneity in the reported variables, a strict comparison between the endoscopic and surgical groups was not possible to perform. In addition, with regard to PD, the use of laparoscopic and robotic-assisted PD is emerging. Retrospective studies showed comparable efficacy with reduced hospital stay and blood loss compared to open PD [94,95]. Nevertheless, recent published evidence failed to show the superiority or even equivalence of the laparoscopic approach to the Whipple procedure [96]. Indeed, a randomised controlled trial was stopped prematurely because of an excess of mortality in the laparoscopic group compared with the open approach [97]. In our meta-analysis, no studies analyzing laparoscopic or robotic-assisted PD could be included but these techniques might be interesting in future treatment of patients with advanced ALs.

To address these unanswered questions, a prospective randomized controlled trial should be initiated. Nevertheless, due to the rarity of ALs and lack of clear stratification attributes, this trial will not be realizable. This call for an international multicenter retrospective study that analyzes EA, SA and PD for AL in detail to provide evidence for therapeutic algorithms and data for the implementation of guidelines in the treatment of different types of ALs, including recurrent or incomplete resected lesions and additional ablative therapies [98].

In conclusion, the present meta-analysis provides an overview about the different therapeutic options for non-invasive ALs, carcinoma in situ and T1-node-negative adenocarcinoma. Our data suggest an improved rate of complete resection in surgical interventions compared to EA. But this benefit was accompanied by a significantly higher rate of complications, in particular after PD. However, high heterogeneity and limited data quality affect the reliability of data and results should be interpreted with caution. Further research is needed to establish clear guideline recommendations for the treatment of ALs.

## Figures and Tables

**Table 1:** Evalulation of the included studies.

Study 1st Author	Year	NOS-Score	Selection I.1	Selection I.2	Selection I.3	Selection I.4	Comparability II	Outcome III.1	Outcome III.2	Outcome III.3
Ahn	2013	**7**	1	1	1	0	1	1	1	1
Alvarez-Sanchez	2017	**7**	1	1	1	0	1	1	1	1
Amini	2014	**8**	1	1	1	1	1	1	1	1
Bellizzi	2009	**6**	1	0	1	1	0	1	1	1
Boix	2009	**5**	1	0	1	1	0	1	1	0
Catalano	2004	**7**	1	1	1	0	1	1	1	1
Ceppa	2013	**9**	1	1	1	1	2	1	1	1
Chang	2014	**6**	1	0	1	1	0	1	1	1
Cheng	2004	**6**	1	0	1	1	0	1	1	1
Chung	2018	**6**	1	1	1	0	1	1	1	0
De Palma	2015	**7**	1	1	1	0	1	1	1	1
Demetriades	2006	**7**	1	0	1	1	1	1	1	1
Desilets	2001	**7**	1	1	1	0	1	1	1	1
Dixon	2005	**6**	1	0	1	0	1	1	1	1
Dubois	2016	**9**	1	1	1	1	2	1	1	1
Gao	2016	**8**	1	1	1	1	1	1	1	1
Grobmyer	2008	**8**	1	1	1	1	1	1	1	1
Harano	2011	**7**	1	1	1	0	1	1	1	1
Hong	2018	**7**	1	0	1	1	1	1	1	1
Hyun	2017	**6**	0	1	1	0	1	1	1	1
Igarashi	2010	**6**	1	0	1	1	0	1	1	1
Irani	2009	**9**	1	1	1	1	2	1	1	1
Ismail	2014	**6**	1	0	1	1	0	1	1	1
Jeanniard-Malet	2011	**6**	1	0	1	1	0	1	1	1
Jung	2009	**5**	1	0	1	1	0	1	1	0
Kahaleh	2004	**4**	1	1	1	0	0	1	0	0
Kang	2017	**6**	1	0	1	1	0	1	1	1
Katsinelos	2006	**6**	1	0	1	1	0	1	1	1
Kim a	2013	**6**	1	0	1	1	0	1	1	1
Kim c	2017	**8**	1	1	1	1	1	1	1	1
Kim b	2011	**8**	1	1	1	1	1	1	1	1
Klein	2018	**9**	1	1	1	1	2	1	1	1
Lee a	2016	**7**	1	1	1	0	1	1	1	1
Lee b	2016	**8**	1	1	1	1	1	1	1	1
Ma	2014	**6**	1	0	1	1	0	1	1	1
Maguchi	2003	**6**	1	1	1	0	0	1	1	1
Mansukhani	2017	**7**	1	0	1	1	1	1	1	1
Napoleon	2014	**7**	1	1	1	0	1	1	1	1
Nguyen	2010	**7**	1	1	1	0	1	1	1	1
Norton	2002	**6**	1	0	1	1	0	1	1	1
Onkendi	2014	**8**	1	1	1	1	1	1	1	1
Ouaïssi	2006	**7**	1	1	1	1	0	1	1	1
Patel	2011	**6**	1	0	1	1	0	1	1	1
Petrone	2013	**7**	1	1	1	0	1	1	1	1
Poincloux	2014	**7**	1	1	1	0	1	1	1	1
Ridtitid	2014	**6**	1	0	1	1	0	1	1	1
Salmi	2012	**7**	1	1	1	0	1	1	1	1
Schneider	2016	**7**	1	1	1	1	1	1	1	0
Shim	2014	**7**	1	1	1	0	1	1	1	1
Stiles	2018	**8**	1	1	1	1	1	1	1	1
Treitschke	1999	**7**	1	0	1	1	1	1	1	1
Tsuji	2015	**6**	1	0	1	1	0	1	1	1
Van der Wiel	2018	**6**	1	0	1	1	0	1	1	1
Will	2013	**6**	1	0	1	1	0	1	1	1
Yamamoto	2018	**7**	1	1	1	0	1	1	1	1
Yamao	2010	**6**	1	0	1	1	0	1	1	1
Yoon	2005	**7**	1	1	1	1	1	1	1	0
Yoon	2007	**8**	1	1	1	1	1	1	1	1
Zadorova	2001	**6**	1	1	1	0	0	1	1	1

Overview over all rated publications in our Systemativ Review, using the Newcastel-Ottawa-Score. Green shows good, yellow mediocre and red low reporting quality.

**Table 2:** Characteristics of the included studies.

Study 1st Author	Year	Country	Patients	Period Start	End	Design	Procedure
Ahn	2013	Korea	retrospective	2006	2009	monocentric	EA
Alvarez-Sanchez	2017	France	retrospective	05/1999	09/2013	monocentric	EA
Amini	2014	USA	retrospective	2004	2010	register, multicentric	EA, SA, PD
Bellizzi	2009	USA	retrospective	NA	NA	monocentric	EA
Boix	2009	Spain	retrospective	01/1995	02/2007	monocentric	EA
Catalano	2004	USA	retrospective	1998	2001	mulitcentric	EA
Ceppa	2013	USA	retrospective	1991	2010	monocentric	EA, SA
Chang	2014	Korea	retrospective	08/2002	06/2011	monocentric	EA
Cheng	2004	USA	retrospective	1994	2003	monocentric	EA
Chung	2018	Korea	retrospective	03/2006	03/2014	monocentric	EA
De Palma	2015	Italy	retrospective	2008	2013	monocentric	EA
Demetriades	2006	Greece	retrospective	1990	2004	monocentric	SA
Desilets	2001	USA	retrospective	1991	2000	monocentric	EA
Dixon	2005	Canada	retrospective	1992	2002	mulitcentric	SA
Dubois	2016	Switzerland	retrospective	2005	2015	monocentric	EA, SA
Gao	2016	China	retrospective	01/2001	12/2014	monocentric	SA, PD
Grobmyer	2008	USA	retrospective	01/1991	12/2006	monocentric	SA
Harano	2011	Japan	retrospective	11/1995	07/2009	monocentric	EA
Hong	2018	Korea	retrospective	2004	2016	monocentric	SA
Hyun	2017	Korea	prospective, randomized	01/2014	12/2015	multicentric	EA
Igarashi	2010	Japan	retrospective	10/2002	03/2009	monocentric	EA
Irani	2009	USA	retrospective	1997	2007	monocentric	EA
Ismail	2014	Finland	retrospective	2000	2012	monocentric	EA
Jeanniard-Malet	2011	France	retrospective	12/2003	11/2008	monocentric	EA
Jung	2009	Korea	retrospective	07/2003	06/2008	monocentric	EA
Kahaleh	2004	USA	retrospective	03/2000	05/2003	monocentric	EA
Kang	2017	Korea	retrospective	01/2007	07/2014	multicentric	EA
Katsinelos	2006	Greece	retrospective	1998	2004	monocentric	EA
Kim a	2013	Korea	retrospective	09/2005	03/2012	monocentric	EA
Kim b	2011	Korea	retrospective	01/1999	12/2008	monocentric	EA, SA
Kim c	2017	Korea	retrospective	01/2001	09/2016	monocentric	EA, SA
Klein	2018	Australia	retrospective	2018	06/2017	monocentric	EA
Lee a	2016	Korea	prospective randomized	01/2012	07/2014	multicentric	EA
Lee b	2016	Korea	retrospective	09/1994	06/2013	monocentric	SA, PD
Ma	2014	USA	retrospective	1999	2010	multicentric	EA
Maguchi	2003	Japan	retrospective	04/1997	10/2002	monocentric	EA
Mansukhani	2017	India	retrospective	2009	2015	monocentric	SA
Napoleon	2014	France	prospective	09/2003	01/2006	mulitcentric	EA
Nguyen	2010	USA	retrospective	NA	NA	monocentric	EA
Norton	2002	USA	retrospective	09/1997	11/1999	monocentric	EA
Onkendi	2014	USA	retrospective	1994	2009	monocentric	EA, SA, PD
Ouaïssi	2006	France	retrospective	1981	2004	monocentric	SA
Patel	2011	USA	retrospective	05/1996	08/2009	monocentric	EA
Petrone	2013	Italy	retrospective	10/2000	06/2009	monocentric	EA
Poincloux	2014	France	retrospective	2004	2011	monocentric	EA
Ridtitid	2014	USA	retrospective	07/1995	07/2012	monocentric	EA
Salmi	2012	France	retrospective	02/2002	12/2008	monocentric	EA
Schneider	2016	Germany	prospective	2001	2014	monocentric	SA
Shim	2014	Korea	retrospective	09/2006	04/2012	monocentric	EA
Stiles	2018	USA	retrospective	2004	2014	register, multicentre	SA, PD
Treitschke	1999	Germany	retrospective	1983	1999	monocentric	SA, PD
Tsuji	2015	Japan	retrospective	05/1999	04/2014	monocentric	EA
Van der Wiel	2018	The Netherlands	retrospective	2002	2016	monocentric	EA
Will	2013	Germany	prospective	NA	NA	multicentric	EA
Yamamoto	2018	Japan	retrospective	05/1999	12/2016	monocentric	EA
Yamao	2010	Japan	retrospective	09/2000	06/2008	monocentric	EA
Yoon	2005	Korea	retrospective	1986	2002	monocentric	PD
Yoon	2007	Korea	retrospective	1996	2006	monocentric	EA, SA
Zadorova	2001	Czech Republic	retrospective	1994	1999	monocentric	EA

EA: endoscopic ampullectomy, SA: surgical ampullectomy, PD: pancreaticoduodenectomy.

**Table 3:** Outcomes of the included studies.

Study 1st Author	Year	Type	Number	% Male	Age (y)	Lesion Size (mm)	R0 (%)	Recurrence %	Follow-Up (m)	Complications Overall	AP	Bleeding	Perforation	Infection	Stenosis	Leakage	Others	% Adenoma	% Adeno-Ca	Hereditary Syndroms	Asymptomatic %
Ahn	2013	EA	43	NA	N	15	74	14	10.4	32.6	16.3	9.3	NA	4.7	2.3	NA	NA	100	0	0	100
Alvarez-Sanchez	2017	EA	28	61	71.3	18	43	58	28	NA	NA	NA	NA	NA	NA	NA	NA	NA	NA	0	100
Bellizzi	2009	EA	45	60	62.9	NA	34.8	73	12	NA	NA	NA	NA	NA	NA	NA	NA	9.1	0	0	NA
Boix	2009	EA	21	43	67.2	NA	28.6	16.7	15.9	23.8	19	4.8	0	NA	0	NA	NA	52.4	47.6	0	0
Catalano	2004	EA	103	48	53.1	22.8	NA	9.7	36	9.7	4.9	1.9	NA	NA	2.9	NA	NA	80.6	5.8	0	NA
Ceppa	2013	EA	68	47	57,8	NA	80	NA	NA	18	9	9	NA	NA	NA	NA	NA	NA	NA	0	NA
Chang	2014	EA	82	65.9	54.7	13	92.7	6.6	24.2	NA	9.8	12.2	0	0	1.2	NA	NA	91.5	NA	0	45
Cheng	2004	EA	55	40	59	NA	73.7	33.3	30	14.5	9.1	29.1	1.8	NA	3.6	NA	NA	100	0	52.4	29.1
Chung	2018	EA	122	53.3	58.2	NA	49.2	12.3	82	NA	13.9	8.2	1,6	4.1	4.9	NA	NA	95.9	2.5	0	52.5
De Palma	2015	EA	27	62.9	68	23	92.6	3.7	18.4	18.5	11.1	7.4	NA	NA	NA	NA	NA	NA	NA	0	25.9
Desilets	2001	EA	41	56	68	24.7	91.3	0	19	4.9	2,4	2.4	0	0	0	0	NA	41.5	36.6	0	NA
Dubois	2016	EA	11	63.6	65	15	NA	NA	NA	9.1	NA	NA	NA	NA	NA	NA	NA	NA	0	13.9	NA
Harano	2011	EA	28	61	66.6	170	79	0	66	NA	7	18	0	7	NA	NA	NA	17	39.3	0	100
Hyun	2017	EA	50	48	60.7	11	66	10.9	12	NA	20	44	2	2	4	NA	NA	100	0	0	100
Igarashi	2010	EA	36	50	66	16.3	NA	8.3	NA	55.6	30	17	0	2.8	5.6	NA	NA	NA	NA	0	100
Irani	2009	EA	102	NA	NA	24	84.3	7.8	NA	20.6	9.8	4.9	2	1	2.9	NA	NA	NA	NA	0	NA
Ismail	2014	EA	61	62.2	NA	NA	52.3	NA	NA	24.6	9.8	18	3.3	NA	NA	NA	NA	78.7	16.4	0	19.7
Jeanniard-Malet	2011	EA	42	54.8	63	NA	92.9	12.1	15	23.8	14.3	7.1	NA	2.4	NA	NA	NA	100	NA	0	61.9
Jung	2009	EA	22	41	58	18.4	54.5	16.7	5.6	22.7	18.2	4.5	4.5	9.1	NA	NA	NA	100	0	0	NA
Kahaleh	2004	EA	56	55	62	22.3	86	NA	NA	NA	7.1	3.6	NA	1.8	NA	NA	NA	NA	NA	0	NA
Kang	2017	EA	104	66	60.5	13.5	89.4	0	44.2	31.7	15.4	17.3	7.7	NA	NA	NA	NA	77.9	5.8	60.6	38.5
Katsinelos	2006	EA	14	57.1	62.6	14.4	78.6	18.2	28.4	14.3	7.1	7.1	0	0	0	NA	NA	100	NA	0	0
Kim a	2013	EA	72	56.9	56.4	8.7	90.3	7.7	23.7	25	8.3	16.7	0	NA	NA	NA	NA	100	NA	0	NA
Kim b	2011	EA	22	NA	NA	NA	86	27	10	50	27.3	13.6	4.5	NA	4.5	NA	NA	NA	NA	0	0
Kim c	2017	EA	10	70	64	8	100	10	15	50	NA	30	10	0	0	0	10	NA	NA	0	NA
Klein	2018	EA	125	50.4	64	20	97.6	17	18.5	NA	7.2	44.8	0.8	NA	NA	NA	NA	NA	NA	37	NA
Lee a	2016	EA	45	55.6	56	NA	84.4	4.4	NA	26.7	15.6	6.7	2.2	0	2.2	NA	NA	NA	NA	0	NA
Ma	2014	EA	26	34.6	27.8	9.9	38.5	58.3	84.5	30.8	19.2	3.8	NA	NA	15.4	NA	NA	NA	NA	34.6	NA
Maguchi	2003	EA	12	58.3	65.7	21.6	100	0	NA	41.7	25	25	8.3	NA	NA	NA	NA	NA	NA	0	NA
Napoleon	2014	EA	93	47.3	57	NA	NA	5.4	36	42	23.7	11.8	4.3	8.6	2.2	NA	NA	NA	NA	0	100
Nguyen	2010	EA	36	52.8	63	23.9	72,2	5.6	NA	NA	0	30.6	NA	NA	NA	NA	NA	88.9	11.1	0	100
Norton	2002	EA	28	54	42	NA	46,2	9.5	13	NA	15.4	7.7	3.8	NA	9.5	NA	NA	NA	NA	0	0
Onkendi	2014	EA	130	52.3	NA	17	NA	32	NA	29	14.6	13.1	0.7	NA	NA	NA	0.7	NA	NA	0	NA
Patel	2011	EA	38	42	54.3	17.3	81	15.8	17.2	15.8	7.9	5.3	0	2.6	0	NA	NA	100	0	0	55.3
Petrone	2013	EA	14	50	74.6	15.3	57,1	57.1	29.6	NA	NA	NA	NA	NA	NA	NA	NA	NA	33.3	73.3	NA
Poincloux	2014	EA	56	57.1	64	16	96,4	21.4	24	19.5	10.7	7.1	1.8	1.8	NA	NA	1.8	76.8	23.2	0	100
Ridtitid	2014	EA	182	48.4	61.4	16.6	73,6	15	190	18.7	3.8	12.6	1.6	NA	3.8	NA	0.5	75.8	6.6	0	39
Salmi	2012	EA	61	45	64	NA	NA	5	36	11	10	5	3	NA	NA	NA	NA	53	16	0	NA
Shim	2014	EA	39	61	62	13.9	74,3	7.7	15	71.8	17.9	10.3	5.1	NA	NA	NA	5.1	81.6	7.7	0	100
Tsuji	2015	EA	115	65.2	64.5	16.2	80,9	NA	NA	NA	10.4	18.2	2.6	1.7	4.3	NA	NA	90.4	9.6	0	NA
Van der Wiel	2018	EA	87	51.7	65	27.7	77	10.7	60	25.3	3.4	12.6	8.1	1.1	0	NA	NA	100	0	0	71
Will	2013	EA	54	46.3	64.6	NA	79,2	11.8	NA	18.5	13	5.6	1.9	NA	NA	NA	NA	44.4	33.3	0	79.6
Yamamoto	2018	EA	177	61	62.7	14.4	74,6	0	30	40.7	10.2	20.3	2.8	2.8	6.8	NA	NA	93.2	6.8	0	NA
Yamao	2010	EA	36	58.3	66	14.3	80,6	3.4	17.9	NA	8.3	8.3	0	0	2.8	NA	NA	100	0	0	100
Yoon	2007	EA	23	NA	NA	18	100	NA	NA	NA	NA	NA	NA	NA	NA	NA	NA	66.2	33.8	0	NA
Zadorova	2001	EA	16	56.3	68	NA	NA	11.1	NA	14.8	7.4	7.4	NA	NA	NA	NA	NA	100	NA	0	100
Amini	2014	SA	41	49	74	17	NA	NA	NA	NA	NA	NA	NA	NA	NA	NA	NA	0	100	0	NA
Ceppa	2013	SA	41	40	56.5	NA	90	NA	NA	42	10	NA	NA	15	NA	NA	17	56	12	0	NA
Demetriades	2006	SA	20	60	68.5	13	100	25	85	10	0	0	0	10	0	0	0	8	12	0	100
Dixon	2005	SA	19	53	64.2	27	63	28	48.6	37	0	0	0	5	0	5	27	63	37	0	100
Dubois	2016	SA	19	37	69	14	NA	NA	NA	68	NA	NA	NA	NA	NA	NA	NA	63	26	5.3	NA
Gao	2016	SA	21	59	68	12	100	31.8	75	13.6	NA	23.8	NA	14.3	NA	NA	0	0	100	0	0
Grobmyer	2008	SA	29	NA	63	20	NA	10	16	45	10	0	0	41	0	14	35	90	14	4	NA
Hong	2018	SA	26	50	60.2	20	100	9.1	72	30.8	0	3.8	0	7.7	3.8	0	15.5	88,5	11,5	0	NA
Kim b	2011	SA	21	NA	NA	NA	NA	4.7	18	23.8	9.5	NA	NA	NA	NA	NA	9.4	38	14,3	0	45
Kim c	2017	SA	21	57.1	61	15	100	0	8	23.8	NA	4.76	0	9.5	4.7	4.7	0	61,9	19	0	29.1
Lee b	2016	SA	18	50	59	16	89	11.1	50	33	0	0	0	23	6	6	0	0	100	0	52.5
Mansukhani	2017	SA	11	NA	56	NA	100	9	34	9	0	0	0	0	0	0	9	64	36	0	25.9
Ouaïssi	2006	SA	26	58	59	20	96	15	86	7.7	0	0	0	0	1	1	0	58	15	30	NA
Schneider	2016	SA	44	36	67	NA	100	8.1	54	36	4.3	NA	NA	3.3	NA	2.2	24.9	100	0	0	NA
Treitschke	1999	SA	36	41	59	NA	NA	0	42	NA	NA	NA	NA	NA	NA	NA	NA	86	0	0	100
Amini	2014	PD	136	52	72	13	NA	NA	NA	NA	NA	NA	NA	NA	NA	NA	NA	0	100	0	100
Gao	2016	PD	22	52	67	12	100	23.8	75	42.8	NA	4.5	NA	9.1	NA	NA	19	0	100	0	100
Lee b	2016	PD	119	46	62	18	98.3	11.8	50	43	0	4	0	7	0	8	17	0	100	0	NA
Stiles	2018	PD	434	55.1	62.9	18	99.1	NA	29.4	NA	NA	NA	NA	NA	NA	NA	NA	0	100	0	19.7
Yoon	2005	PD	67	58	56.7	18	95.5	18.2	58.9	49.3	NA	6	0	16.4	0	19.4	13.4	0	100	0	61.9

**Table 4:** Technical specifications of endoscopic ampullectomy.

Study 1st Author	Year	EUS Performed	Patients with EUS (%)	Prior ERCP	Paitents with Prior ERCP (%)	Implantation of Stents	Submucosal Injection	Additional Intervention
Ahn	2013	YES	100	YES	100	PD	YES	EA
Alvarez-Sanchez	2017	YES	100	YES	100	NA	NA	Surg
Bellizzi	2009	NO	0	NO	0	NO	NO	NA
Boix	2009	YES	NA	NA	NA	NO	YES	APC
Catalano	2004	YES	NA	NA	NA	BD + PD	NO	Surg + RFA
Ceppa	2013	NA	NA	NA	NA	NO	NO	NA
Chang	2014	YES	45	YES	100	BD + PD	YES	Surg
Cheng	2004	YES	29.1	NA	NA	PD	YES	NA
Chung	2018	YES	52.5	YES	100	BD + PD	YES	Surg + APC
De Palma	2015	YES	25.9	YES	100	PD	NO	NA
Desilets	2001	NO	0	YES	100	BD + PD	YES	APC
Dubois	2016	NA	NA	NA	NA	NO	NO	NA
Harano	2011	YES	100	YES	100	BD + PD	YES	EA
Hyun	2017	YES	100	NA	NA	BD + PD	YES	Surg
Igarashi	2010	YES	100	YES	100	BD + PD	NO	APC
Irani	2009	YES	42	NA	NA	NA	NA	NA
Ismail	2014	YES	19.7	NA	NA	PD	NO	NA
Jeanniard-Malet	2011	YES	61.9	NO	0	BD + PD	NO	Surg
Jung	2009	NO	0	YES	100	BD + PD	YES	Surg
Kahaleh	2004	YES	NA	NO	0	PD	NO	NA
Kang	2017	YES	38.5	YES	79.8	BD + PD	YES	EA + Surg
Katsinelos	2006	NO	0	YES	100	BD + PD	YES	Surg
Kim a	2013	NO	0	NO	0	PD	NO	NA
Kim b	2011	NA	NA	NA	NA	NA	NA	NA
Kim c	2017	NA	NA	NA	NA	NA	NA	NA
Klein	2018	YES	NA	YES	100	BD + PD	YES	Surg
Lee a	2016	YES	NA	YES	NA	PD	NO	EA + Surg
Ma	2014	NO	0	NA	NA	BD + PD	YES	EA + Surg
Maguchi	2003	NO	0	NO	0	BD + PD	NO	NA
Napoleon	2014	YES	100	NO	0	PD	YES	RFA
Nguyen	2010	YES	100	NO	0	PD	NO	Surg
Norton	2002	NO	0	YES	100	BD + PD	NO	Surg
Onkendi	2014	YES	27.8	YES	49.4	NA	NA	NA
Patel	2011	YES	55-3	YES	100	PD	NO	NA
Petrone	2013	NO	0	YES	100	PD	NO	APC
Poincloux	2014	YES	100	YES	100	PD	YES	Surg + APC + RCT
Ridtitid	2014	YES	39	YES	100	PD	YES	Caut
Salmi	2012	NA	NA	NA	NA	BD + PD	YES	EA
Shim	2014	YES	100	YES	100	BD + PD	YES	EA+APC
Tsuji	2015	NO	0	NO	0	NO	NO	Surg
Van der Wiel	2018	YES	71	YES	100	PD	YES	APC
Will	2013	YES	79.6	YES	44.8	NA	NA	NA
Yamamoto	2018	YES	NA	NA	NA	NO	NO	NA
Yamao	2010	YES	100	YES	100	BD + PD	NO	APC
Yoon	2007	NA	NA	NA	NA	NA	NA	NA
Zadorova	2001	YES	100	YES	93.6	BD + PD	NO	APC

## Supplementary Materials

The following are available online at https://www.mdpi.com/2077-0383/9/11/3622/s1. Supplemental Material and methods.

## Abbreviations

AA	ampullary adenoma
AC	ampullary adenocarcinoma
AE	adverse event
AL	ampullary lesion
APC	Argon plasma coagulation
CI	confidence interval
EA	Endoscopic ampullectomy
ESAP	Endoscopic versus Surgical Ampullectomy vs Pancreaticoduodenectomy
FAP	Familial adenomatous polyposis
GIST	Gastrointestinal stroma tumor
MeSH	medical subject headings
NOS	Newcastle-Ottawa-Scale
PD	Pancreaticoduodenectomy
PICO(S)	Population Intervention Comparison Outcome Study
PRISMA	Preferred Reporting Items for Systematic reviews and Meta-Analyses
RFA	Radiofrequency ablation
SA	Surgical ampullectomy
