# Supplementary material for: Systematic Review with Meta-Analysis: Endoscopic and Surgical Resection for Ampullary Lesions
Source: J Clin Med. 2020 Nov 10;9(11):3622. doi: 10.3390/jcm9113622 (PMC7696506; doi:10.3390/jcm9113622)
Supplement: Supplementary file 1 [file jcm-09-03622-s001.pdf]

Supplemental Material and methods

strategy

We used medical subject headings (MeSH) and keywords including “endoscopic ampullectomy”, “endoscopic papillectomy”, “surgical ampullectomy” or “ampullary tumor”. The complete search term was: “ampullectomy” OR “papillectomy” OR “endoscopic ampullectomy” OR “surgical ampullectomy” OR “trans-duodenal ampullectomy” OR “endoscopic papillectomy” OR “ampulloma” OR “ampullary tumor” OR “ampullary tumour” OR “papillary tumor” OR “papillary tumour” OR “ampullary adenoma” OR “ampullary adenocarcinoma” OR „periampullary tumor“ OR „periampullary tumour“ OR „periampullary adenoma“ OR „periampullary adenocarcinoma“ OR „cancer of ampulla of Vater“. The search was restricted to English, German and French literature. Our search strategy was based on the PICOS model [92]: P (Population: all patients with ALs), I (Intervention: rate of complete resection (R0)), C (Comparison: endoscopic interventions compared to surgery), O (Outcome: is endoscopy non-inferior to surgery), S (Study type: retrospective or prospective interventional and observational studies).

Screening and data extraction

All titles and abstracts of the search were independently screened by two reviewers (MH and CH) and checked for eligibility. All eligible papers were evaluated by two reviewers (EAA and FA for endoscopic papers; CH, AG and MH for surgical papers). The calculated agreement was substantial (Kappa=0.677). In case of disagreement, consent was resolved by discussion (CH and MH) and reasons for exclusion were registered. Blinding was not performed. If there were any duplicates, the latest publication was used for analysis.

The following data were extracted (Tables 1 and 2): study design, origin, publication year, interventions (EA, SA, PD), patient number, gender, age, size of the lesion, percentage of R0, recurrence, follow-up, complications, mortality, differentiation of adenoma and T1 adenocarcinoma from other histology, sporadic or familiar lesions, clinical presentation (asymptomatic vs symptomatic) and technical specificities.
